# A Remotely Delivered, Semaglutide-Supported Specialist Weight Management Program: Preliminary Findings From a Retrospective Service Evaluation

**DOI:** 10.2196/53619

**Published:** 2023-12-28

**Authors:** Rebecca Richards, Gina M Wren, Peta Campion, Michael Whitman

**Affiliations:** 1 Second Nature London United Kingdom; 2 Nuffield Department of Primary Care Health Sciences University of Oxford Oxford United Kingdom

**Keywords:** digital health intervention, smartphone, obesity management, medication, mobile phone

## Abstract

**Background:**

Digital weight management interventions have the potential to increase access to novel pharmacotherapy for people living with obesity. At present, there is limited real-world evidence on the effectiveness, feasibility, and acceptability of this type of intervention.

**Objective:**

This retrospective service evaluation examines real-world data to evaluate the preliminary impact of Second Nature’s 24-month, remotely delivered, semaglutide-supported weight management intervention for adults living with obesity at 12 weeks.

**Methods:**

Retrospective data were extracted in October 2023 for participants who started the intervention between June 8, 2023, and July 22, 2023. The primary outcomes were weight change (kg) and percentage of weight change at 12 weeks. The secondary outcomes were the proportion of participants who achieved ≥5% and ≥10% weight loss and the feasibility and acceptability of this type of intervention. Descriptive statistics were used to evaluate the baseline characteristics, retention, engagement, prevalence of side effects, and weight change. A paired 2-tailed *t* test was used to determine the significance of weight change. Content analysis was used to analyze the free-text questionnaire responses.

**Results:**

A total of 113 participants with a mean baseline BMI of 38.4 kg/m^2^ (SD 7.3) were included in the analysis (n=102, 90.4% women, mean age 46.6, SD 11.1 years). Over 12 weeks, 23% (n=26) of participants withdrew from the intervention. A total of 70.8% (n=80) of participants provided weight data at 12 weeks. The average weight loss observed over this 12-week period was 6.5 (SD 4.4) kg (*P*<.001) or 6.4% (SD 4.2%) of their starting weight (*P*<.001). Of the 80 participants who recorded weight readings, 62.5% (n=50) achieved ≥5% weight loss, and 11.3% (n=9) achieved ≥10% weight loss. Engagement with the app-based program declined from a mean of 131 (SD 142.6) home screen views in week 0 to 35 (SD 57.1) in week 11. Common side effects reported over 12 weeks included feeling more tired than usual, constipation, and feeling sick. However, a significant proportion of participants reported no side effects. Most participants (n=106, 93.8%) did not experience any difficulties in medication administration. Qualitative data showed that most participants had a positive or neutral experience of the intervention, with some reporting perceived benefits as early as 4 weeks. Most participants did not feel that improvements in the intervention were needed; however, some participants faced issues with medication shipping or logistics.

**Conclusions:**

This retrospective preliminary service evaluation suggests that a remotely delivered semaglutide-supported weight management intervention has the potential to be effective, feasible, and acceptable for self-paying consumer adults with obesity in the United Kingdom. Areas for further improvement were highlighted, including user engagement in an app-based program. A full-service evaluation at the end of the 24-month intervention with a larger sample size is required to support these early findings.

## Introduction

### Background

Obesity is characterized by excess body fat and an increased risk of various physical and mental comorbidities, such as type 2 diabetes, cancer, cardiovascular disease, anxiety, and depression, as well as increased overall mortality risk [1-3]. This chronic, relapsing condition is a major public health issue in the United Kingdom, where projections suggest the annual cost of overweight and obesity to the National Health Service (NHS) will reach £9.7 billion (US $12.3 billion) by 2050 and broader societal expenses are anticipated to reach £49.9 billion (US $63.4 billion) [4]. Historically, the foundation of obesity treatment has been time-limited behavioral weight loss programs that are delivered by a coach in a group setting, focusing on dietary change and increasing physical activity [5]. Such interventions have been shown to be successful in reducing body weight in the best cases by approximately 5% to 10% in the short term, which is associated with improved health outcomes and quality of life [6,7]. However, sustained weight loss with behavioral interventions is poor because of the failure to address the physiological factors that drive obesity [8].

In February 2022, a novel pharmacotherapy treatment, semaglutide, was approved by the National Institute for Health and Care Excellence (NICE) as a promising adjunct therapy for behavioral weight management in people with obesity, defined as a BMI of ≥35 kg/m^2^ with at least 1 weight-related condition, such as type 2 diabetes [9]. Semaglutide, a glucagon-like peptide-1 receptor agonist (GLP-1RA), mimics the action of the hormone GLP-1, which helps regulate appetite, prevent overeating by improving the satiety signal from the gut to the brain, and regulate blood sugar levels by stimulating insulin production and slowing down the digestion of food [10,11]. By addressing the physiological factors that dysregulate appetite, people with obesity are more easily able to make the necessary behavioral changes, such as dietary changes and increased physical activity, to support weight loss. A randomized controlled trial of semaglutide (once weekly injections of 2.4 mg) and lifestyle modification showed that people with obesity lost approximately 12% more weight compared with lifestyle modification alone [12], and people lost and sustained, on average, 15% of their body weight over a 2-year period using this combination treatment [13].

The NICE guidelines specified that treatment with semaglutide should be provided as part of a specialist weight management service with oversight from a multidisciplinary team (MDT), such as within a tier 3 or tier 4 specialist service [9]. However, specialist weight management services in the United Kingdom, which are traditionally delivered in person, are limited and vary geographically owing to a lack of funding [14]. Furthermore, existing services face increasing problems such as long waiting lists, understaffing, and a lack of treatment flexibility [14-16], which can result in treatment delays and adversely affect patient outcomes [15]. Consequently, many people eligible for semaglutide cannot access this treatment. In recognition of this issue and to increase access to this treatment, in June 2023, the UK government announced a £40 million (US $50,255,907) 2-year pilot to assess the feasibility and effectiveness of remotely delivered semaglutide treatment alongside behavioral weight management [17]. This was followed by the launch of an early value assessment by NICE in August 2023 of expanding specialist weight management services outside of hospital settings using digital weight management providers [18].

To date, only one published study has examined the remote delivery of a specialist weight management service that included patients who used weight loss medication [19]. This study evaluated 169 patients with obesity (BMI ≥45 or ≥40 kg/m^2^ with a comorbidity) who were selected from a choice of dietary interventions, including diet and lifestyle goal setting, calorie counting, a low-carbohydrate diet, a partial meal replacement plan, or intermittent fasting. The findings showed that remote delivery of this intervention was equally effective and acceptable as face-to-face support. However, only a small proportion of these patients were prescribed weight loss medications, with only 6.5% receiving GLP-1RAs. Further research is therefore needed to demonstrate the effectiveness, feasibility, and acceptability of remotely delivered semaglutide and behavioral weight management treatments outside hospital settings.

### Objectives

This service evaluation aimed to evaluate the preliminary impact of a remotely delivered semaglutide-supported specialist weight management intervention for self-paying adults living with obesity in the United Kingdom, including the feasibility and acceptability of this intervention. The primary outcomes were weight change (kg) and percentage of weight change at 12 weeks. The secondary outcomes were the proportion of participants who achieved ≥5% and ≥10% weight loss and the feasibility and acceptability of this type of intervention.

## Methods

### Overview

This service evaluation used a retrospective analysis of preliminary data from self-paying consumers. The Health Research Authority decision tool was used to determine the nature of this study [20]. This study met the criteria for service evaluation, as outlined by the Health Research Authority and Medical Research Council: (1) it was designed and conducted solely to assess current care; (2) it evaluated our current service without reference to a standard; (3) it involved an intervention already in use; (4) it involved analysis of existing data; (5) there was no allocation to the intervention; and (6) there was no randomization [21]. Furthermore, the study only involved the use of previously collected, anonymized, and non-NHS data that could not be traced back to identifiable individuals. When registering for the intervention, participants were required to provide consent for their anonymized data to be collected for research purposes, including analysis and publication. As per the General Data Protection Regulations, participants could request to have their information deleted at any time. A privacy policy detailing data collection, storage, and processing is hosted on the Second Nature website [22].

### Ethics Approval

This study was not considered research by the NHS and, therefore, did not require ethical approval (Multimedia Appendix 1).

### Participants

Retrospective data were extracted from Second Nature’s database in October 2023 and were deidentified. Participants included in this analysis were self-paying consumers who started the intervention, provided by Second Nature Health Habits Ltd, between June 8, 2023, and July 22, 2023, and completed 12 weeks.

To participate in the program, the participants completed a comprehensive questionnaire assessment of their suitability for semaglutide. This information was reviewed by the customer support team, and the participants were contacted for any missing information. The assessment was then reviewed for approval by an independent prescribing pharmacist. Eligible participants were adults aged 18 to 75 years living with obesity (BMI ≥30 kg/m^2^), with access to and the ability to use a smartphone or tablet device. Exclusion criteria included present or past history of eating disorders, pregnancy, breastfeeding or actively trying to conceive, specific allergies to any ingredient in the medicine or excipients, use of certain medications (eg, diabetes medication or medications for conditions highlighted in the exclusion criteria), and presence of any of the following health conditions: presence or history of thyroid cancer, active cancer, inflammatory bowel disease (eg, Crohn disease or ulcerative colitis), celiac disease, chronic malabsorption syndrome, pancreatitis, impaired liver, kidney disease, heart failure, multiple endocrine neoplasia type 2, gallbladder problems, or diabetic retinopathy.

### Intervention

#### Overview

Semaglutide was prescribed and dispensed by independent prescribing pharmacists with suitable competencies employed by Pharmalogic Chemist, a national leader in specialist NHS and private pharmacy services in the United Kingdom. An app-based weight management program was provided by Second Nature, a leading digital weight management provider in the United Kingdom. Once deemed clinically suitable, the medication was dispatched directly to the participants’ preferred address. Each participant also received a leaflet of information on the medication, a sharps bin for the safe disposal of injection needles, a recipe book, wireless weighing scales (Renpho Elis 1 Smart Body Scale), and access to Second Nature’s app-based weight management program. Participants were supported through an intervention by a designated health coach. The health coaches were dietitians (registered with the Health and Care Professions Council [HCPC]) or nutritionists (registered with the Association for Nutrition). When a participant was coached by a nutritionist, a dietitian provided supervision.

In total, 2 senior members of the Second Nature team created a comprehensive training program for health coaches, which included (1) an overview of semaglutide (how it works, indications, contraindications, side effects, and how to advise on side effects); (2) the Second Nature program and content; (3) scope of practice, clinical scenarios (including management of nutrition experience, weight changes, dosage, and injections) and MDT communication (such as when and how to escalate side effects to the prescribing team); and (4) safeguarding, including protocols for allergic reactions, disordered eating, misuse of medication, extreme weight loss, and other considerations, such as new pregnancy, breastfeeding, cancer diagnosis, or insulin use. The lead prescriber was then taken through a copy of the protocols for approval, with the view that the MDT would communicate and work together to iterate the protocols as needed in real time. Once approved, all health coaches underwent training across 4 video call sessions delivered by a senior dietitian, which were recorded for future reference.

The MDT consisted of a dietitian (registered with the HCPC) with a BSc in sport science and biology and MSc in human nutrition; a nutritionist (registered with the Association for Nutrition) with a BSc in applied sports nutrition and MSc in applied sports nutrition; a health coach manager (an HCPC registered dietitian) with a BBiomedSci in nutrition and metabolism in human health and master of health sciences in nutrition and dietetics, and a clinical pharmacist (MPhram, registered with the General Pharmaceutical Society). A safeguarding lead (HCPC registered dietitian) was also on hand, where needed, including conducting coaching quality reviews (fortnightly assessments and feedback).

#### App-Based Program

Second Nature’s semaglutide-supported digital weight management program is up to 24 months in duration, accessed by smartphone or web-based app, and consists of five phases: (1) Prepare (week 0), where participants are taught about using the medication and program; (2) Adapt (weeks 1-17), where participants build a support group through peer and coach interactions, receive support for side effects, and acquire knowledge of nutrition and habit formation; (3) Grow (weeks 18-34), where participants gain a deeper understanding of their new behaviors and cement their new habits; (4) Transition (weeks 35-51), where participants prepare for life beyond medication by learning to self-regulate their behaviors and eating habits; and (5) Maintain (week 52+), where participants practice the skills they have learned to maintain their weight loss after they stop the medication. From 52 weeks, participants can continue to receive medication and support and have access to all app features; however, they do not receive any new educational content. Following the completion of the program, participants retain their access to the program and resources indefinitely.

The program covers a range of topics to support sustainable weight loss through medication and lifestyle changes. Throughout each phase, participants are provided with structured education, tailored for use with semaglutide medication, on nutrition guidelines, managing side effects, the psychology of eating, forming habits, physical activity, sleep, stress management, and improving mental well-being. The program is designed to provide the practical solutions participants need to form a healthy lifestyle, as well as explore the *why* behind their behaviors so that they have the ability to reflect and self-regulate their habits in the future.

The program was developed by an MDT of medical doctors, psychologists, dietitians, nutritionists, and behavioral scientists in line with relevant NICE guidance for obesity management and behavior change [23-27]. Behavior change techniques and insights were also adopted from the “Behavior Change Wheel” [28], with new behaviors encouraged through self-monitoring, goal setting, social rewards, and education from credible sources. Previous studies have shown that the Second Nature Weight Management Program (without GLP-1RA medication support) is effective for weight loss for adults with overweight, obesity, and type 2 diabetes [29-32].

The features of the program include daily educational articles and goal setting, weight, steps and sleep tracking, and a toolbox of resources (further educational materials, recipes, meal planners, journals, food diaries, and guided exercise videos). Each participant was provided with 1 to 1 tailored guidance through private, text-based communication with their health coach as and when they requested. Health coaches were available during normal working hours from Monday to Friday. In addition to coach support, participants received peer support from a group chat feature (supervised by their health coach) that they entered together upon joining the program. On Mondays, Wednesdays, and Fridays, health coaches would post a message in the group chat feature. On Tuesdays and Thursdays, coaches would respond to any messages.

Engagement with the app was monitored automatically, and health coaches were alerted if a participant did not log a home screen view (HSV) by week 1 day 1 (the start of the Adapt phase) or did not complete their monthly mandatory web-based check-in questionnaire or weigh-in. If a participant did not complete their check-in or weight reading on time, email reminders would be sent, followed by messages from their health coach and, finally, a call from the customer support team. This would help participants obtain their next medication dose on time.

#### Medication

Due to the delay in the launch of Wegovy (semaglutide) in the United Kingdom at short notice, participants were prescribed Ozempic (semaglutide) for off-label use as an alternative in the interim. This is an evidence-based and widely accepted clinical practice in which licensed medicine is unavailable [12,13]. Participants were informed of this change and given the opportunity to ask further questions. In light of the supply limitations of Ozempic at 1 mg, treatment dosage titrations were revised accordingly. Participants were started on a dose of 0.25 mg and escalated to 0.5 mg, where appropriate, and were maintained at this dose. This was to ensure that existing clinical benefits and patient safety were maintained in light of the national shortage. On July 18, 2023, the Department of Health and Social Care issued a National Patient Safety Alert to address the shortage of GLP-1 receptor agonists. New initiation of Ozempic was strongly discouraged, and a deadline of October 18, 2023, to stop prescribing this medication was issued. In response to this alert, we stopped accepting new consumer signups to our program and began exploring alternative treatment pathways for existing users.

Procedures were in place to ensure the safe and effective use of semaglutide, with particular attention to dosage, injections, and safeguarding of participants. Upon sign-up, participants received a PDF that contained information on semaglutide and video instructions on how to administer it (from the manufacturer’s site) [33]. There were also instructions for administering the medication added to a medication “Toolbox” in the Second Nature app and information on the medication was also included in daily articles as part of the app program. If participants needed further information or help, they could contact their health coach directly via the chat feature in the app, and both prescribers and pharmacists were made available as and when needed.

Throughout the intervention period, the participants were required to complete a mandatory monthly web-based questionnaire to report any side effects, which was reviewed by the prescribing team. This information was used to determine the continuation of semaglutide prescription by an independent prescribing pharmacist. The pharmacist had the autonomy to engage directly with participants for any clarifications or further inquiries based on this information. In addition, participants could raise experiences of side effects at any time via private chats with their health coach in the app. Health coaches would respond within 24 hours (excluding weekends), and reported side effects were shared with the prescribing team through a cloud-based instant messaging service, which was reviewed by the pharmacist. The instant messaging service was also used to raise safeguarding issues, issues related to low BMI, or excessive weight loss. The pharmacist would respond within at least 4 hours and provide advice for the health coach to provide feedback to the participant or call the participant directly if required. The outcomes of any calls were reported back to the health coach via the instant messaging service and noted on the participant’s profile on the Second Nature coaching platform as well as clinical and pharmacy records. As part of each monthly check-in, participants were required to use their weighing scales to share a weight reading with the app via Bluetooth.

This iterative process of health coaches communicating side effects and receiving advice from pharmacists enabled the teams to codevelop a protocol for the management of side effects. Because of this iterative process, the side effects were later classified into mild, moderate, and severe categories. For mild side effects, health coaches provided prespecified advice on lifestyle modifications such as encouraging smaller, more frequent meals, slow and mindful eating, avoiding certain foods or drinks, staying hydrated, and managing sleep routines. If the patient was still struggling after 1 week, this was escalated to the prescribing team for support. For moderate to severe side effects, health coaches would escalate immediately to the prescribing team for advice. The pharmacist would then determine whether they would need to call the participant or give advice to the health coach who would then pass this to the participant. In case of emergencies, such as allergic reactions, health coaches would instruct participants to contact emergency services via telephoning 999 or visiting the hospital.

### Data Collection and Measures

#### Overview

Preliminary data were collected from the participants who had undergone the first 12 weeks of the 24-month intervention. Participant characteristics (height, age, and gender) and contact details were collected via an onboard questionnaire. These data were saved to Second Nature’s database and viewed on the coaching platform. Information was checked by the customer support team in the case of missing information. To verify participant identity and ensure access to accurate data to safely prescribe medication, background checks were performed via a third-party service called LexisNexis. Details were shared via a secure software interface (name, gender, date of birth, and address), and a status report was returned in the response from the LexisNexis interface. Users are categorized as “pass,” “fail,” or “refer” based on matches from databases, such as the Electoral Roll, Companies House, Experian, and Equifax. In total, 2 positive matches were necessary for a “pass.” Inconsistencies in birth data, deceased status, or age below 18 years resulted in an instant “fail.” Weight readings were transferred from the wireless weighing scales to the app via Bluetooth and saved to Second Nature’s database.

#### Effectiveness

The primary outcomes were weight change (kg) and percentage weight change 12 weeks after starting the program. The secondary outcomes included the proportion of participants who achieved ≥5% and ≥10% weight loss. Weight readings at baseline and the lowest weight reading between 76 and 104 days from the participant’s start date for the intervention were extracted for analysis. This date range was carefully selected to ensure a consistent 12-week evaluation period across all participants, allowing for both standardized follow-up and flexibility in data collection. Recognizing potential variations in scheduled check-ins and the real-world challenges participants might face, this range ensured data collection close to the 12-week mark while maximizing the retention of usable data. The participants were sent wireless weighing scales to transfer their weight data to Second Nature. To ensure accurate and consistent measurements, accompanying instructions advised placement on a firm, flat surface, weighing first thing in the morning after using the rest room on the same day at the same time each week. The readings were automatically transmitted to Second Nature’s database after use. To ensure accuracy, a weight validation algorithm was used, which only accepted measurements within a predicted range, considering the last recorded weight and the time since the last reading. In cases of irregular readings, an email alert was sent to the participant to explain that the reading would not be saved. If this was deemed a mistake, participants could contact their health coach or email their support team. This methodology aimed to eliminate abnormal readings (such as those from other household members) to ensure reliable data for analysis.

#### Feasibility

The secondary outcomes included the feasibility of the intervention, assessed using the following measures.

##### Retention and Cancellation Reasons

Withdrawal was defined as participants cancelling their subscription to the Second Nature program. The reasons for cancellation were collected using the app. This information was automatically retrieved from the app and saved in Second Nature’s secure database.

##### Engagement

Data related to user engagement were automatically collected while users interacted with the intervention over the 12 weeks observed in this study and were saved in Second Nature’s secure database. We tracked each participant’s HSVs on both the web and smartphone apps to measure engagement with the intervention. These events acted as indicators of overall application use and were recorded each time the participant opened the app. This information was retrieved in conjunction with baseline and weight data using Metabase, an open-source analytics tool.

##### Side Effects

Participants were emailed a mandatory web-based check-in questionnaire every 4 weeks throughout the intervention period to collect information on medication side effects. Participants had the option to select from a drop-down menu of 15 possible side effects, “None” or “Other.” Selecting “Other” prompted a mandatory free-text response for the description. This preliminary analysis included data from 4-, 8-, and 12-week questionnaires.

#### Acceptability

The secondary outcomes included the acceptability of the intervention, assessed using the following measures.

##### Medication Administration

Responses to the following two questions from the mandatory web-based check-in questionnaire at 4, 8, and 12 weeks were extracted to assess the acceptability of medication administration: (1) “Where on your body have you been injecting the medication?” which allowed for free-text responses, and (2) “Are you having any difficulty injecting yourself?” which had 3 preset answers: “No,” “Yes, and it means I’m not injecting once a week on the same day,” and “Yes, but I’m still injecting once a week on the same day.”

##### Participant Experience

Responses to the following two questions from the mandatory web-based check-in questionnaire at 4, 8, and 12 weeks were extracted to assess the acceptability of the program: (1) “How have you found the last few weeks of the program?” and (2) “What do you think we could have done better so far to improve your experience on the program?” Participants provided free-text responses to both questions.

### Statistical Analysis

Descriptive statistics were used to evaluate the baseline characteristics of the study population, including retention, engagement, prevalence of side effects, and weight change. The primary objective was to determine whether the weight change observed over a span of 12 weeks was statistically significant from the baseline. A paired 2-tailed *t* test was used, as the interest spanned both potential deviations from the null hypothesis mean of 0, encompassing both possible weight gain and losses. Statistical analysis was performed using R open-source statistical language through the R-Studio interface, and the criterion for statistical significance was *P*<.05.

### Qualitative Analysis

Content analysis was conducted to analyze the free-text responses to the mandatory web-based check-in questions. For each question, at each time point, the responses were coded and organized into higher-level categories by MW. For example, a positive experience was characterized by explicit positive statements or implicit indications of a positive experience or outcome; a neutral experience was characterized by statements that implied a satisfactory outcome, without explicit positive or negative experiences or outcomes; and perceived benefits were characterized by explicit benefits or positive outcomes related to the intervention, such as reduced appetite, improved relationship with food, increased awareness of portion sizes, easier-to-make dietary changes, and weight loss. Participants’ responses were organized into more than one category. The codes and categories were then discussed with RR and refined. Finally, the frequency of occurrence of each category was calculated.

## Results

### Baseline Characteristics

In total, 113 participants were included in the analysis (Table 1). Most participants (n=102, 90.3%) were women, and the average age was 46.6 (SD 11.1) years. The mean baseline BMI was 38.4 (SD 7.3) kg/m^2^.

**Table 1:** Baseline characteristics of participants (N=113).

Characteristics	Values
Age (y), mean (SD)	46.7 (11)
BMI (kg/m^2^), mean (SD)	38.4 (7.3)
Weight (kg), mean (SD)	105 (19.8)
Women, n (%)	102 (90.3)

### Effectiveness

Of the 113 participants who started the intervention, 70.8% (n=80) provided weight data at 12 weeks. The average weight loss observed over this 12-week period was 6.5 kg (SD 4.4; *P*<.001) or 6.4% of their starting weight (SD 4.2; *P*<.001). Of the 80 participants who recorded weight readings, 62.5% (n=50) achieved ≥5% weight loss, and 11.3% (n=9) achieved ≥10% weight loss.

### Feasibility

#### Retention and Cancellation Reasons

Over 12 weeks, 23% (n=26) of participants withdrew from the intervention. Of the remaining 77% (n=87) of participants who remained active, 7 did not submit weight readings at 12 weeks. Of those who withdrew, 65% (17/26) shared reasons for cancellation, which were categorized as follows: unmanageable side effects (n=6), financial reasons (n=7), lack of progress or reasons related to medication shortage and supply issues (n=3), and no longer eligible (n=1).

#### Engagement

**Figure 1:**
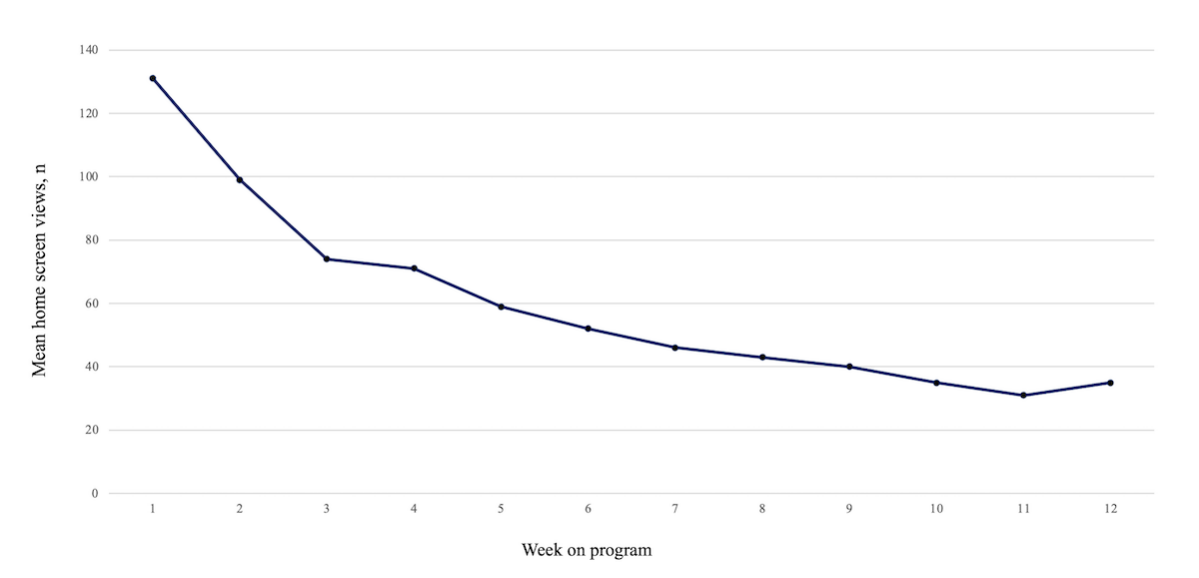
Mean count of app home screen views by week on program.

Figure 1 shows the mean number of HSVs per week. The mean HSV started at 131 (SD 142.6) in week 0 and displayed a mostly linear decline to 35 (SD 57.1) in week 11.

A total of 75.4% (n=86) participants completed their mandatory check-in questionnaire at 4 weeks, 68.4% (n=78) completed it at 8 weeks, and 60.5% (n=69) completed it at 12 weeks. A variety of side effects were reported over the 12-week study period (Table 2). At 4 weeks, the most commonly reported side effects were “feeling more tired than usual” (38/86, 44%), “constipation” (20/86, 23%), and “feeling sick” (19/86, 22%), and 29% (25/86) reported no side effects at this stage. At 8 weeks, 42% (33/78) of participants reported no side effects, and the most common side effects remained “feeling sick” (18/78, 23%), “feeling more tired than usual” (17/78, 22%), and “constipation” (15/78, 19%). At 12 weeks, half of the participants (35/69, 51%) reported no side effects, and the most common side effects were “constipation” (16/69, 23%), “feeling sick” (12/69, 17%), and “heartburn or indigestion” (11/69, 16%). Across the 12 weeks, “other” was selected 10 times by the participants.

**Table 2:** Prevalence of side effects at 4, 8, and 12 weeks.

Side effect	4 weeks (n=86), n (%)	8 weeks (n=78), n (%)	12 weeks (n=69), n (%)
Feeling more tired than usual	38 (44)	17 (22)	8 (12)
None	25 (29)	33 (42)	35 (51)
Feeling sick	19 (22)	18 (23)	12 (17)
Constipation	20 (23)	15 (19)	16 (23)
Heartburn or indigestion	12 (14)	10 (13)	11 (16)
Diarrhea	9 (10)	4 (5)	4 (6)
Headaches	7 (8)	7 (9)	2 (3)
Feeling dizzy	2 (2)	1 (1)	0 (0)
Fever or high temperature	0 (0)	0 (0)	0 (0)
Vomiting	1 (1)	1 (1)	1 (1)
Tender or swollen stomach	2 (2)	0 (0)	2 (3)
Severe stomach pain	1 (1)	1 (1)	0 (0)
Feeling anxious	1 (1)	1 (1)	2 (3)
Yellowing of the skin or eyes	0 (0)	0 (0)	0 (0)
Reactions in the injection site	0 (0)	0 (0)	0 (0)
Hair loss	0 (0)	2 (3)	5 (7)
Other	3 (4)	6 (8)	1 (1)

### Acceptability

#### Medication Administration

Participants reported injecting the medication in their triceps, abdomen, and thighs.

Most participants (n=106, 93.8%) reported no difficulties with medication administration. At the 4-week check-in, only 1 participant reported having difficulty injecting themselves. This participant was included in the total of 3 participants who reported having difficulties injecting themselves during the 8-week check-in period. Finally, 4 participants who had not previously reported difficulties injecting themselves at weeks 4 and 8 reported difficulties in injecting themselves at the 12-week check-in. Only 1 participant stated that difficulties with injecting themselves meant that they were not injecting once a week on the same day, as prescribed. All other participants stated that they were still injecting themselves once a week on the same day, despite the difficulties.

#### Participant Experience

Free-text responses to the survey question, “How have you found the last few weeks of the program?” were classified into 19 categories (Table 3). Most participants reported positive experiences at 4 and 8 weeks (60/86, 70% and 45/78, 58%, respectively); however, this was reduced at 12 weeks (29/69, 42%). The second most common experience at 4, 8, and 12 weeks was neutral, reported by 21% (18/86), 26% (20/78), and 38% (26/69) of participants, respectively. Approximately 20% (17/86) of participants reported benefits from the intervention at 4 weeks; however, benefits were only mentioned in response to this question by a small number of participants at weeks 8 and 12 (3/78, 4% and 3/69, 4%, respectively).

**Table 3:** Categories of free-text responses to the survey question, “How have you found the last few weeks on the program?” at 4-, 8- and 12-week check-ins.

Categories	4 weeks (n=86), n (%)	8 weeks (n=78), n (%)	12 weeks (n=69), n (%)
Positive experience	60 (70)	45 (58)	29 (42)
Neutral experience	18 (21)	20 (26)	26 (38)
Intervention benefits	17 (20)	3 (4)	3 (4)
Change in appetite	12 (14)	14 (18)	10 (15)
Adapting to the program	4 (5)	1 (1)	0 (0)
No change in appetite	2 (2)	2 (3)	1 (1)
Illness	2 (2)	0 (0)	0 (0)
Other priorities	1 (1)	7 (9)	6 (9)
Technical issues with the app	1 (1)	0 (0)	0 (0)
Insufficient information	1 (1)	0 (0)	0 (0)
Not started the medication	1 (1)	0 (0)	0 (0)
Suggestions for program improvements	1 (1)	0 (0)	0 (0)
Not engaged with the program	1 (1)	0 (0)	0 (0)
Reduced medication effectiveness	0 (0)	0 (0)	6 (9)
Confusion about eligibility	0 (0)	0 (0)	1 (1)
Slow progress	0 (0)	3 (4)	4 (6)
Weight loss plateau	0 (0)	2 (3)	4 (6)
Negative experience	0 (0)	2 (3)	0 (0)

A minority of participants reported noticing changes in their appetite at each of the 4-, 8-, and 12-week check-ins (12/86, 14%, 14/78, 18% , and 10/69, 15%, respectively). At the 4- and 8-week check-ins, most responses indicated a reduction in appetite, a minority of participants perceived no change in appetite, and a small number of participants perceived fluctuations in appetite. At week 8, one participant reported an increased appetite. The 12-week check-in highlighted a shift in appetite experiences, in which most appetite-related responses were about experiencing an increase in appetite.

At the 12-week check-in, 9% (6/69) mentioned that other priorities (such as work, holidays, school holidays, and other time pressures) were barriers to engagement in the intervention, 9% (6/69) perceived the medication to have reduced in effectiveness, 6% (4/69) reported they felt their weight loss progress was slow, and 6% (4/69) reported that they had experienced a weight loss plateau in the last few weeks.

Free-text responses to the survey question, “'What could we improve on the program?” were classified into 23 categories (Table 4). At the 4-week check-in, nearly half of the participants (42/86, 49%) felt that no improvements to the program were necessary. This increased to 59% (46/78) of the participants at week 8 and then decreased to 33% (23/69) at week 12. The second most common response at week 4, which was to improve shipping or logistics of medication, was reported by 17% (15/86) of the participants; however, this was only reported by 7% (5/69) of the participants at week 12. Across the 4-, 8-, and 12-week check-ins, a minority of participants had suggestions to improve the content of the program (8/86, 9%, 9/78, 12%, and (8/69, 12%, respectively), such as more information before signing on to the program, the ability to choose a start date, fixing technical issues, and additional information on nutrition. At the 12-week check-in, 13% (9/69) expressed concerns about medication supply, specifically around the inability to increase their dose because of shortages. Furthermore, a small number of participants (9/69, 13%) reported suggestions for additional support, highlighting the need for more personalized interactions, such as 1-to-1 video calls with their coach, and more frequent communication with the coach. Finally, 9% (6/69) of the participants felt that group engagement could be improved.

**Table 4:** Categories of free-text responses to the survey question, “What could we improve on the program?” at 4-, 8- and 12-week check-ins.

Categories	4 weeks (n=86), n (%)	8 weeks (n=78), n (%)	12 weeks (n=69), n (%)
No improvements necessary	42 (49)	46 (59)	23 (33)
Shipping and logistics	15 (17)	8 (10)	5 (7)
Content suggestions	8 (9)	9 (12)	8 (12)
Confusion about medication	8 (9)	0 (0)	0 (0)
Support suggestions	5 (6)	4 (5)	9 (13)
Group engagement	2 (2)	4 (5)	6 (9)
Technical issues	2 (2)	3 (4)	0 (0)
Syncing with other apps	1 (1)	0 (0)	0 (0)
App improvements	1 (1)	0 (0)	0 (0)
Medication supply	0 (0)	2 (3)	9 (13)
Font too small	0 (0)	1 (1)	0 (0)
Medication logging	0 (0)	1 (1)	0 (0)
Overwhelmed	0 (0)	1 (1)	0 (0)
Holiday	0 (0)	1 (1)	0 (0)
More side effects check-ins	0 (0)	1 (1)	0 (0)
Communication suggestions	0 (0)	1 (1)	0 (0)
Program cost	0 (0)	0 (0)	3 (4)
Communication updates	0 (0)	0 (0)	2 (3)
Move to nonmedication program	0 (0)	0 (0)	1 (1)
Spare medication or needles	0 (0)	0 (0)	1 (1)
Option to stay on current dose	0 (0)	0 (0)	1 (1)
Slow progress	0 (0)	0 (0)	1 (1)
More frequent check-ins	0 (0)	0 (0)	1 (1)

## Discussion

### Principal Findings

To increase access to novel pharmacotherapy for adults with obesity outside hospital settings, NICE and the UK government are facilitating a pilot evaluation and early value assessment of remotely delivered semaglutide and behavioral weight management [17,18]. Currently, there is a lack of evidence regarding the effectiveness, feasibility, and acceptability of this digital approach [18]. We presented preliminary findings from a service evaluation of a remotely delivered semaglutide-supported specialist weight management intervention for self-paying adults living with obesity in the United Kingdom to contribute to this evidence base. This intervention was shown to be effective for weight loss at 12 weeks and feasible, as almost three-quarters of the participants were retained and engagement was high at the beginning. Importantly, medication administration and overall intervention, including an app-based program, were found to be acceptable to participants. Areas highlighted for improvement included user engagement in the app-based program, medication shipping and logistics, and the management of side effects. Overall, these preliminary findings suggest that a remotely delivered semaglutide-supported weight management intervention has the potential to be effective, feasible, and acceptable for self-paying adults living with obesity in the United Kingdom.

### Comparison With Prior Work

Our preliminary findings are comparable with those of a similar observational study of injectable semaglutide in a real-world setting that showed an average of 6.6% body weight loss at 12 weeks for adults with obesity treated at outpatient clinics [34]. The percentages of participants who achieved ≥5% and ≥10% body weight loss were also comparable [34]. Furthermore, our findings align with those observed in randomized controlled trials, such as the global phase 3 Semaglutide Treatment Effect in People with Obesity trial, which showed an average body weight loss of approximately 6% at 12 weeks into the 68-week intervention for people with obesity [12]. The side effect profile in this study was also similar to that found in real-world and randomized controlled trials [12,34,35]; however, a key difference was that tiredness appeared to be a more prevalent self-reported side effect in this study. Future qualitative research is required to provide insights into this finding. Overall, these findings suggest that a remotely delivered semaglutide-supported app-based weight management intervention can be as effective as in person and outpatient support, as well as the results obtained under optimal trial conditions.

The acceptance of a remotely delivered medication-supported weight management intervention for adults with obesity is a novel finding of this study. Currently, only one published study has explored the acceptability of this type of intervention in adults with obesity; however, only approximately 6% of the participants used GLP-1RA medication [19]. Therefore, this study offers preliminary evidence to suggest that this type of intervention is acceptable as a specialist weight management treatment for people with obesity; however, further evaluation is needed at 24 months to determine the acceptability of the full length of treatment for a specialist weight management intervention. The findings of this study also highlighted potential barriers to remote treatment with semaglutide, including issues with shipping or logistics in receiving medication. The finding that participants desired and valued coaches and community interaction throughout the intervention is reflected in the broader literature on the useful components of digital weight management interventions [36]. In addition, the finding that engagement with the app-based program declined over time reflects a body of literature suggesting that this is a key issue with this type of intervention [37]. Further research is needed to understand the factors that impacted user engagement and the relationship between engagement and weight loss outcomes [37]. This preliminary analysis of acceptability indicates that this novel information can now be used to iterate and improve the interventions to avoid ongoing issues for participants and potentially improve treatment outcomes.

### Limitations

This study has some limitations that should be considered. This study was a service evaluation that used retrospective data and did not include a control group. Therefore, it is not possible to draw definite conclusions about the effectiveness of the intervention, and the results should be interpreted cautiously. However, our findings are comparable with those from real-world and randomized clinical trials of this type of intervention for weight loss achieved at 12 weeks [12,34,35]. The study design also meant that a small number of participants did not submit weight readings within the specified data collection period, thus limiting the sample size. Due to the commercial nature of the evaluation, data on ethnicity and socioeconomic status were not collected, which limited conclusions on the acceptability of the intervention. Further research with a larger sample size and longer follow-up at 24 months is required to support these preliminary findings.

Owing to medication shortages, participants in this study received up to 0.5 mg of semaglutide over 12 weeks, instead of up to 1.7 mg at 12 weeks, as prescribed for adults with obesity by the Medicines and Healthcare Products Regulatory Agency and NICE guidance [9]. Nevertheless, the weight loss findings were comparable to those of studies that used a higher dose of semaglutide [11,12,34].

Most of the sample was made up of women, and participants were self-paying consumers; therefore, they may have been more motivated to lose weight than a nonconsumer sample. The findings of this are therefore limited in their generalizability to semaglutide for adults with obesity referred from the NHS; however, this sample is largely representative of enrollment in private weight management services [38].

Finally, other relevant outcome data, such as clinical outcomes (eg, hemoglobin A_1c,_ blood pressure, and lipid profile), psychological outcomes, and quality of life, were not collected. A definitive, fully powered, randomized controlled trial with more time points and longer-term follow-up is needed to determine the impact of our intervention on these health outcomes and its wider economic impact.

### Conclusions

This retrospective preliminary service evaluation suggests that a remotely delivered semaglutide-supported weight management intervention has the potential to be effective, feasible, and acceptable for self-paying consumer adults with obesity in the United Kingdom. Areas for further improvement were highlighted, including user engagement in an app-based program, medication logistics, and the management of side effects. A full evaluation at 24 months with a larger sample size is required to confirm these early findings.

## Appendix

Multimedia Appendix 1 Health Research Authority decision tool result.

## Abbreviations

GLP-1RA: glucagon-like peptide-1 receptor agonist
HCPC: Health and Care Professions Council
HSV: home screen view
MDT: multidisciplinary team
NHS: National Health Service
NICE: National Institute for Health and Care Excellence

## References

[1] Wharton S, Lau DC, Vallis M, Sharma AM, Biertho L, Campbell-Scherer D, Adamo K, Alberga A, Bell R, Boulé N, Boyling E, Brown J, Calam B, Clarke C, Crowshoe L, Divalentino D, Forhan M, Freedhoff Y, Gagner M, Glazer S, Grand C, Green M, Hahn M, Hawa R, Henderson R, Hong D, Hung P, Janssen I, Jacklin K, Johnson-Stoklossa C, Kemp A, Kirk S, Kuk J, Langlois MF, Lear S, McInnes A, Macklin D, Naji L, Manjoo P, Morin MP, Nerenberg K, Patton I, Pedersen S, Pereira L, Piccinini-Vallis H, Poddar M, Poirier P, Prud'homme D, Salas XR, Rueda-Clausen C, Russell-Mayhew S, Shiau J, Sherifali D, Sievenpiper J, Sockalingam S, Taylor V, Toth E, Twells L, Tytus R, Walji S, Walker L, Wicklum S (2020). Obesity in adults: a clinical practice guideline. CMAJ.

[2] Whitlock G, Lewington S, Sherliker P, Clarke R, Emberson J, Halsey J, Qizilbash N, Collins R, Peto R, Prospective Studies Collaboration (2009). Body-mass index and cause-specific mortality in 900 000 adults: collaborative analyses of 57 prospective studies. Lancet.

[3] Abdullah A, Peeters A, de Courten M, Stoelwinder J (2010). The magnitude of association between overweight and obesity and the risk of diabetes: a meta-analysis of prospective cohort studies. Diabetes Res Clin Pract.

[4] (2017). Health matters: obesity and the food environment. Public Health England.

[5] Kirk SF, Penney TL, McHugh TL, Sharma AM (2012). Effective weight management practice: a review of the lifestyle intervention evidence. Int J Obes (Lond).

[6] Hartmann-Boyce J, Johns DJ, Jebb SA, Summerbell C, Aveyard P, Behavioural Weight Management Review Group (2014). Behavioural weight management programmes for adults assessed by trials conducted in everyday contexts: systematic review and meta-analysis. Obes Rev.

[7] Ryan DH, Yockey SR (2017). Weight loss and improvement in comorbidity: differences at 5%, 10%, 15%, and over. Curr Obes Rep.

[8] Hall KD, Kahan S (2018). Maintenance of lost weight and long-term management of obesity. Med Clin North Am.

[9] (2023). Semaglutide for managing overweight and obesity. National Institute for Health and Care Excellence.

[10] Gabery S, Salinas CG, Paulsen SJ, Ahnfelt-Rønne J, Alanentalo T, Baquero AF, Buckley ST, Farkas E, Fekete C, Frederiksen KS, Helms HC, Jeppesen JF, John LM, Pyke C, Nøhr J, Lu TT, Polex-Wolf J, Prevot V, Raun K, Simonsen L, Sun G, Szilvásy-Szabó A, Willenbrock H, Secher A, Knudsen LB, Hogendorf WF (2020). Semaglutide lowers body weight in rodents via distributed neural pathways. JCI Insight.

[11] Friedrichsen M, Breitschaft A, Tadayon S, Wizert A, Skovgaard D (2021). The effect of semaglutide 2.4 mg once weekly on energy intake, appetite, control of eating, and gastric emptying in adults with obesity. Diabetes Obes Metab.

[12] Wilding JP, Batterham RL, Calanna S, Davies M, Van Gaal LF, Lingvay I, McGowan BM, Rosenstock J, Tran MT, Wadden TA, Wharton S, Yokote K, Zeuthen N, Kushner RF (2021). Once-weekly semaglutide in adults with overweight or obesity. N Engl J Med.

[13] Garvey WT, Batterham RL, Bhatta M, Buscemi S, Christensen LN, Frias JP, Jódar E, Kandler K, Rigas G, Wadden TA, Wharton S (2022). Two-year effects of semaglutide in adults with overweight or obesity: the STEP 5 trial. Nat Med.

[14] Coulton V, Dodhia S, Ells L, Blackshaw J, Tedstone A (2015). National mapping of weight management services: provision of tier 2 and tier 3 services in England. Public Health England.

[15] Hazlehurst J, Logue J, Parretti HM, Abbott S, Brown A, Pournaras DJ, Tahrani AA (2020). Developing integrated clinical pathways for the management of clinically severe adult obesity: a critique of NHS England policy. Curr Obes Rep.

[16] Watkins R, Swancutt D, Alexander M, Moghadam S, Perry S, Dean S, Sheaff R, Pinkney J, Tarrant M, Lloyd J (2023). A qualitative exploration of patient and staff experiences of the receipt and delivery of specialist weight management services in the UK. Patient.

[17] (2023). New drugs pilot to tackle obesity and cut NHS waiting lists. United Kingdom Government.

[18] (2023). Digital technologies for delivering specialist weight-management services to manage weight-management medicine: early value assessment. National Institute for Health and Care Excellence.

[19] Huntriss R, Haines M, Jones L, Mulligan D (2021). A service evaluation exploring the effectiveness of a locally commissioned tier 3 weight management programme offering face-to-face, telephone and digital dietetic support. Clin Obes.

[20] Is my study research?. National Health Service Health Research Authority.

[21] (2022). Defining research. Health Research Authority.

[22] Privacy policy. Second Nature.

[23] (2014). Obesity: identification, assessment and management. National Institute for Health and Care Excellence.

[24] (2014). Weight management: lifestyle services for overweight or obese adults. National Institute for Health and Care Excellence.

[25] (2015). Preventing excess weight gain. National Institute for Health and Care Excellence.

[26] (2014). Behaviour change: individual approaches. National Institute for Health and Care Excellence.

[27] (2012). Obesity: working with local communities. National Institute for Health and Care Excellence.

[28] Michie S, van Stralen MM, West R (2011). The behaviour change wheel: a new method for characterising and designing behaviour change interventions. Implement Sci.

[29] Ross JA, Barron E, McGough B, Valabhji J, Daff K, Irwin J, Henley WE, Murray E (2022). Uptake and impact of the English National Health Service digital diabetes prevention programme: observational study. BMJ Open Diabetes Res Care.

[30] Kar P, Goward C, Whitman M, Davies M, Willner T, Shaw K (2020). Engagement and effectiveness of digitally enabled behavioural change support for people living with type 2 diabetes. Pract Diabetes.

[31] Idris I, Hampton J, Moncrieff F, Whitman M (2020). Effectiveness of a digital lifestyle change program in obese and type 2 diabetes populations: service evaluation of real-world data. JMIR Diabetes.

[32] Hampton J, Allen E, Edson C (2017). Service evaluation of a digital behavioural change programme. Future Healthc J.

[33] How to use the Wegovy® Pen. Second Nature YouTube page.

[34] Alabduljabbar K, Alsaqaaby M, Neff KJ, Crotty M, le Roux CW (2023). Weight loss response in patients with obesity treated with injectable semaglutide in a real-world setting. Endocrine.

[35] Wadden TA, Bailey TS, Billings LK, Davies M, Frias JP, Koroleva A, Lingvay I, O'Neil PM, Rubino DM, Skovgaard D, Wallenstein SO, Garvey WT (2021). Effect of subcutaneous semaglutide vs placebo as an adjunct to intensive behavioral therapy on body weight in adults with overweight or obesity: the STEP 3 randomized clinical trial. JAMA.

[36] Khaylis A, Yiaslas T, Bergstrom J, Gore-Felton C (2010). A review of efficacious technology-based weight-loss interventions: five key components. Telemed J E Health.

[37] Sharpe EE, Karasouli E, Meyer C (2017). Examining factors of engagement with digital interventions for weight management: rapid review. JMIR Res Protoc.

[38] Gudzune KA, Doshi RS, Mehta AK, Chaudhry ZW, Jacobs DK, Vakil RM, Lee CJ, Bleich SN, Clark JM (2015). Efficacy of commercial weight-loss programs: an updated systematic review. Ann Intern Med.

